# Pediatric Rhabdomyosarcoma: Epidemiology and Genetic Susceptibility

**DOI:** 10.3390/jcm10092028

**Published:** 2021-05-09

**Authors:** Bailey A. Martin-Giacalone, P. Adam Weinstein, Sharon E. Plon, Philip J. Lupo

**Affiliations:** 1 Department of Pediatrics, Section of Hematology-Oncology, Baylor College of Medicine, Houston, TX 77030, USA; Bailey.Martin-Giacalone@bcm.edu (B.A.M.-G.); Phillip.Weinstein@bcm.edu (P.A.W.); splon@bcm.edu (S.E.P.); 2 Program in Translational Biology and Molecular Medicine, Graduate School of Biomedical Sciences, Baylor College of Medicine, Houston, TX 77030, USA; 3 Genetics and Genomics Graduate Program, Graduate School of Biomedical Sciences, Baylor College of Medicine, Houston, TX 77030, USA; 4 Department of Molecular and Human Genetics, Baylor College of Medicine, Houston, TX 77030, USA

**Keywords:** pediatric cancer, epidemiology, genetic susceptibility, cancer predisposition

## Abstract

Rhabdomyosarcoma (RMS) is the most common soft-tissue sarcoma in children, yet little is known about its etiology. Studies that examine either environmental exposures or germline genetic predisposition in RMS have begun to identify factors that contribute to this malignancy. Here, we summarize epidemiological reports of RMS incidence in terms of several factors, including age at diagnosis, biological sex, and geographic location. We then describe findings from association studies, which explore the role of parental exposures, birth and perinatal characteristics, and childhood exposures in RMS. Further, we discuss RMS predisposition syndromes and large-scale sequencing studies that have further identified RMS-associated genes. Finally, we propose future directions of study, which aim to advance our understanding of the origin of RMS and can provide knowledge for novel RMS therapies.

## 1. Introduction

Soft-tissue sarcomas account for 7% of all pediatric cancer diagnoses in the United States (U.S.) [1], and approximately 50% of all pediatric soft-tissue sarcoma diagnoses are rhabdomyosarcoma (RMS) [2]. RMS has an incidence of approximately 4.71 per million children and adolescents less than 20 years of age in the United States. This is similar to other sarcomas, including osteosarcoma (5.09 per million) and Ewing sarcoma (2.95 per million), but lower than the most frequent pediatric cancer—acute lymphoblastic leukemia (ALL, 32.59 per million) [3]. Importantly, survival for RMS is poor. In the most recently completed Children’s Oncology Group (COG) intermediate-risk study, the four-year event-free survival was 63% [4]. For individuals with metastatic disease, the three-year event-free survival is less than 20%, despite multi-agent therapies [5].

RMS can occur anywhere in the body, including the head and neck, genitourinary organs, extremities, and abdomen. Although still debated, evidence suggests that RMS arises from the mesenchymal cell lineage, which is typically fated to become skeletal muscle tissue [6]. The expressions of myogenic factors are the primary support for this hypothesis [7,8,9]. However, recent reports have shown that RMS can also arise from endothelial progenitors, which suggests a mechanism for tumors that originate in areas that are devoid of skeletal muscle tissue [10].

RMS is traditionally classified into two major histological subtypes, embryonal RMS (ERMS) and alveolar RMS (ARMS), of which 60–70% of cases are ERMS and 20–30% are ARMS [11]. Currently, the World Health Organization (WHO) also recognizes two other less common subtypes, namely, pleomorphic and spindle cell/sclerosing RMS [12]. Notably, 80% of ARMS cases are defined by a chromosomal translocation between *PAX3* or *PAX7* and *FOXO1* genes; these translocations result in fusion genes that largely drive oncogenic activity. The other 20% of ARMS cases are similar to ERMS in terms of clinical outcomes and the pattern of somatic mutations [13]. There is an overwhelming consensus among experts that fusion status is a stronger prognostic factor for risk stratification compared to tumor histology. Therefore, fusion status is now integrated into risk stratification for treatment protocols [13,14,15].

Despite the clinical significance of RMS, the underlying etiologies remain unclear. Evidence supports that environmental and genetic factors individually contribute to this malignancy. Therefore, in this review, we present the current knowledge of the factors that play a role in RMS.

## 2. Epidemiology of RMS

A primary objective of epidemiology is to study the distribution and determinants of disease in human populations. Assessments evaluating the distribution of disease (e.g., incidence, overall survival) are considered descriptive epidemiology, whereas studies evaluating the determinants of disease (e.g., environmental exposures, nutritional status) are considered analytic epidemiology. One of the most comprehensive assessments of the distribution and survival of RMS using a large population-based sample was published in 2009 using data from the Surveillance, Epidemiology, and End Results (SEER) program [16]. The analysis spanned from 1975 to 2005 and included nine U.S. cancer registries, which together represent approximately 35% of the U.S. population; these registries ascertain >98% of cases in their area. However, it is important to note that studies evaluating the determinants of RMS are much more limited, which is likely due to the rarity of this tumor (there are approximately 350 newly diagnosed RMS cases per year in the United States). Additionally, these studies often present equivocal or relatively modest findings. In this section, we summarize studies that have evaluated the distribution and/or determinants of RMS.

### 2.1. Trends in Incidence

Between 1975 and 2005, there was no significant change in the incidence of RMS overall or ERMS in the United States [11]. This is in contrast with the steady increases in the incidences observed for other pediatric cancers [3,17,18]. While there has been an increase in the incidence of ARMS (annual percent change (APC) = 4.20, 95% confidence interval (CI) = 2.60–5.82), this is likely due to changes in diagnostic criteria. While there is no conclusive evidence that the incidence of RMS has changed over time, there are data suggesting that the incidence of RMS varies significantly by demographic factors, including age, biological sex, and geographic location.

### 2.2. Incidence by Age and Sex

**Figure 1:**
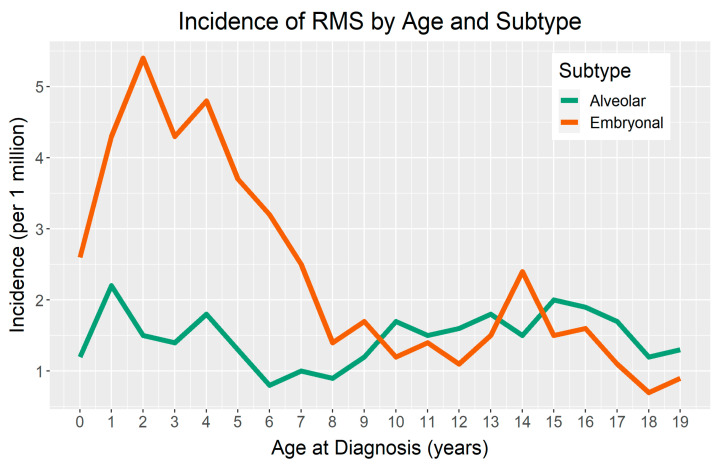
Age-incidence curve of rhabdomyosarcoma (RMS) in terms of the major histological subtypes: embryonal RMS (ERMS) and alveolar RMS (ARMS). The data represent the incidences from 2000–2017 and was published by the Surveillance, Epidemiology, and End Results (SEER) program.

There is a demonstrated difference in the incidence of RMS by age, with peaks occurring during early childhood. For children four years of age or younger, RMS incidence is 6.5 per million in the United States [11]. Notably, there appears to be a second, less pronounced peak in incidence for ERMS during adolescence (Figure 1) [2,11,19]. Osteosarcoma and Ewing sarcoma also illustrate this age-related, second peak in incidence. Valberg et al. suggested that an accelerated pubertal growth period may play a role in susceptibility to sarcomas during adolescence [20].

The incidence of RMS also varies by biological sex. Specifically, RMS incidence is higher in males compared to females (rate ratio (RR) = 1.37, 95% CI = 1.21–1.56); this phenomenon is driven by the predominance of male ERMS diagnoses (RR = 1.51 male to female, 95% CI = 1.27–1.80) [11]. Notably, this disparity (male predominance in risk) is consistent with most other pediatric cancers [21,22], which could yield insights into the etiologies of these malignancies. 

### 2.3. Global Incidence

**Figure 2:**
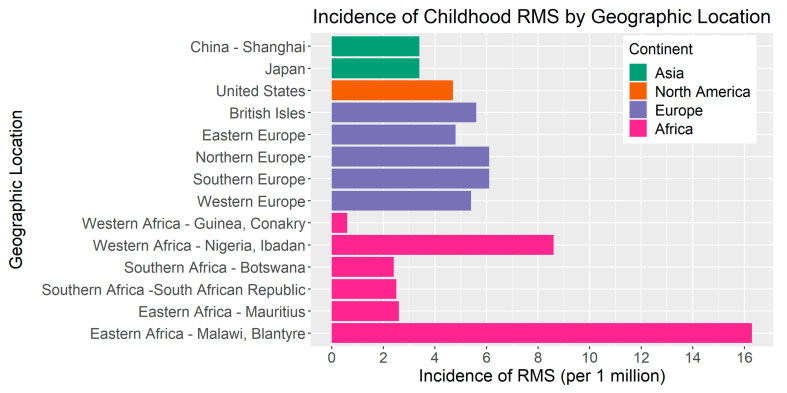
Incidence of RMS per 1,000,000 children by geographic location. Bar color indicates geographic location by continent. RMS incidence in Africa is presented as two countries per region, which illustrate the minimum and maximum values of the range by region, as reported by Stefan et al. [27].

The RMS incidence is modestly elevated in European countries compared to the United States (Figure 2). Based on a report including 59 cancer registries from 19 European countries [23], the overall incidence of RMS in Europe was 5.4 cases per million in children <15 years of age from 1978–1997. The incidence ranged from 4.8 per million in Eastern Europe to 6.1 per million in both Northern Europe and Southern Europe. In a separate study from 1984 to 2010, the incidence of RMS in Sweden was 4.9 cases per million [24]. In contrast, parts of Asia appear to have a lower incidence of RMS compared to the United States and Europe. For instance, in a study of the Shanghai Cancer Registry, the incidence of RMS was 3.4 per million between 2002 and 2005 [25]. Further, a comparative study of the incidence of pediatric cancers in England and Japan estimated that the RMS incidence from 1993–2010 was 3.4 per million (95% CI = 2.8–3.9) in Japan compared to 5.0 per million in England (95% CI = 4.6–5.3) [26]. 

In sub-Saharan Africa, the RMS incidence is highly variable. For example, a study of 16 population-based registries within the African Cancer Registry Network estimated that the incidence rate of childhood RMS in Western Africa ranged from 0.6 to 8.6 per million compared to 2.4 to 2.5 per million in Southern Africa and 2.6 to 16.3 per million in Eastern Africa. The differences in incidence between and within regions may be due to the variability in environmental factors, cancer surveillance, or diagnostic practices [27]. There are ongoing efforts to improve cancer registration efforts and access to diagnostics in low- and middle-income countries [28].

### 2.4. Incidence by Race and Ethnicity

Despite the literature showing racial disparities in other pediatric cancers, there is little evidence to suggest that the incidence of RMS varies by race/ethnicity [17]. For instance, Ognjanovic et al. estimated that the overall incidence of RMS for White children was 4.6 per one million person-years compared to 4.9 in Black children [11]. This is notably different than Ewing sarcoma, where the incidence is low among Black children compared to White children [29]. 

## 3. Non-Genetic Risk Factors

More than half of all RMS cases occur before 10 years of age [11], which indicates that in utero and early-life environmental exposures may play a large role in RMS etiology. Here, we discuss prenatal exposures, birth characteristics, and childhood exposures that may be associated with RMS risk (Table 1).

### 3.1. Parental Exposures

#### 3.1.1. Parental Age

Young and advanced parental age is a suggested risk factor for several pediatric cancers. However, RMS study findings have been equivocal in relation to this factor. In a large assessment including linked data from birth certificates and cancer registries from five U.S. states, each five-year increase in maternal age was modestly associated with RMS risk, even after adjusting for paternal age (adjusted odds ratio (aOR) = 1.19, 95% CI = 1.05–1.34). The association of a five-year increase in paternal age was not significant after adjusting for maternal age (aOR = 1.00, 95% CI = 0.90–1.11) [30]. Most recently, in a study of 198 RMS cases, Lupo et al. reported no significant associations of RMS with maternal age or paternal age. For ERMS, risk was slightly reduced for paternal age ≥ 35 years at delivery (incidence rate ratio (IRR) = 0.51, 95% CI = 0.27–0.97) [31].

#### 3.1.2. Additional Parental Exposures

Some reports have evaluated the role of parental exposures on the risk of RMS in the offspring. These exposures include recreational drug use [32,33], prenatal diagnostic radiation [34], and various occupational exposures [35,36]. For example, a case–control study involving 319 RMS cases found that any maternal X-ray examination during pregnancy was associated with an increased risk of RMS in their children (OR = 1.9, 95% CI = 1.1–3.4). The risk of RMS was greatest for X-ray exposure in the first trimester, with a 5.7-fold increase (95% CI = 1.2–27.8), and specifically, for the association of X-ray exposure in the first trimester and ERMS (OR = 10.5, 95% CI = 1.5–458.4) [34]. Additionally, a recent study that examined 1923 RMS cases from the U.K. National Registry of Childhood Tumors found that paternal occupational exposure to electromagnetic fields was associated with RMS risk in the fathers’ children (OR = 1.67, 95% CI = 1.22–2.28) [37]. However, these associations have yet to be replicated.

### 3.2. Perinatal and Birth Characteristics

#### 3.2.1. Birth Weight and Birth Term

Ognjanovic et al. analyzed data from 583 RMS cases and reported that high birth weight, measured for each 500 g increase, was associated with an increase in the odds of RMS overall (OR = 1.18, 95% CI = 1.09–1.29) and ERMS (OR = 1.27, 95% CI = 1.14–1.42) [38]. In contrast, a study of 722 pediatric RMS cases in California showed that high birth weight, measured as >4000 g, was not associated with RMS overall nor with either histological subtype [39]. Similarly, Lupo et al. did not find a significant association between high birth weight, measured as ≥4000 g, and the odds of overall RMS (OR = 1.35, 95% CI = 0.77–2.37) [40]. However, high birth weight and low birth weight (<2500 g) were significant for ARMS (high: OR = 2.41, 95% CI = 1.09–5.35; low: OR = 4.46, 95% CI = 1.41–14.1).

Neither Ognjanovic et al. nor Shrestha et al. found any significant associations of preterm birth (≤36 weeks of age) and the risk of RMS [38,41]. Conversely, a recent study reported that preterm birth (<37 weeks) was significantly associated with RMS incidence (IRR = 1.74, 95% CI = 1.08–2.79, *p* = 0.02) [31]. Based on these assessments, there is no clear association between birth weight or term and RMS, as there is with some other pediatric cancers, including hepatoblastoma and ALL [42,43].

#### 3.2.2. Birth Defects

Both chromosomal and non-chromosomal birth defects are relatively well-established risk factors for pediatric cancer [44,45]. In a 1995 study of 249 RMS cases, the cases had greater odds of birth defects compared to the controls (OR = 2.36, 95% CI = 0.92–6.52, *p* = 0.05), and this association was significant, specifically in males (OR = 3.16, 95% CI = 1.02–10.41, *p* = 0.02) [46]. However, in a recent study of the risk of cancer in children with birth defects, Lupo et al. assessed 418 cases of RMS and did not find any significant associations with any major chromosomal or non-chromosomal birth defects [44]. The evidence for birth defects and RMS is less clear than for other pediatric cancers, suggesting more work is needed to elucidate these relationships.

### 3.3. Exposures during Childhood

#### 3.3.1. Immune-Related Factors

Immune-related factors are emerging as significant exposures that may contribute to RMS. A report that evaluated allergies and atopy found significant inverse associations with other atopic conditions, such as allergies (OR = 0.60, 95% CI = 0.41–0.87) and hives (OR = 0.61, 95% CI = 0.38–0.97) [40]. Further, the protective relationship of vaccinations against pediatric cancer is also gaining interest. A study of the association of immunizations and the risk of RMS showed that having any incomplete immunizations significantly increased the risk of RMS (OR = 5.30, 95% CI = 2.47–11.33) [47]. Interestingly, this report did not find any significant associations between infections and childhood RMS.

Breast milk is a known source of immune-related components, such as antibodies and prebiotics. Breastfeeding for ≥12 months was significantly associated with a decreased risk of childhood RMS (OR = 0.36, 95% CI = 0.18–0.70). Further, the analysis showed that there was a statistically significant association between increasing breastfeeding duration and a decreased risk of RMS (*p* = 0.01) [40]. 

#### 3.3.2. Additional Childhood Exposures

There is a lack of knowledge of the associations between childhood environmental exposures and RMS. One case–control study from 1982 found that children with diets that included organ meats were at a significantly greater risk of RMS (RR = 3.7, 95% CI = 1.5–8.3, *p* = 0.04). They also reported that children exposed to chemicals other than pesticides and rodenticides were at a 3.2-fold greater risk of RMS (95% CI = 1.1–9.2). However, this study only included 33 RMS cases [48]. Compared to other pediatric cancers, there is a dearth of studies that evaluated the impact of exposures during early life and the risk of RMS.

**Table 1:** Summary of suggested risk factors for pediatric rhabdomyosarcoma.

Risk Factor	Odds Ratio (95% CI)	No. of Cases	References
Parental exposures			
Parental age			
Each 5-year increase in maternal age	1.19 (1.05–1.34) *	556	[30]
Recreational drug use			
Maternal	3.1 (1.4–6.7) *	322	[32]
Paternal	2.0 (1.3–3.3) *	322	[32]
Prenatal diagnostic radiation	1.9 (1.1–3.4) *	319	[34]
Occupational exposures			
Agent Orange exposure	1.72 (0.55–5.41)	319	[36]
Electromagnetic fields	1.67 (1.22–2.28) *	1923	[37]
Perinatal/birth characteristics			
Birth weight			
Overall RMS ^a^, each 500 g increase	1.18 (1.09–1.29) *	583	[38]
ERMS ^b^, each 500 g increase	1.27 (1.14–1.42) *	363	[38]
ARMS ^c^, ≥4000 g	2.41 (1.09–5.35) *	66	[49]
ARMS, <2500 g	4.46 (1.41–14.1) *	66	[49]
Preterm birth			
Overall RMS, GA ^d^ <37 weeks	1.74 (1.08–2.79) *^,e^	198	[31]
ERMS, GA <37 weeks	1.97 (0.98–3.94) ^e^	198	[31]
Childhood exposures			
Allergies	0.60 (0.41–0.87) *	322	[40]
Hives	0.61(0.38–0.97) *	322	[40]
Incomplete immunizations	5.30 (2.47–11.33) *	322	[47]
Breastfeeding, ≥12 months	0.36 (0.18–0.70) *	322	[40]

* Statistically significant, ^a^ rhabdomyosarcoma (RMS), ^b^ embryonal RMS (ERMS), ^c^ alveolar RMS (ARMS), ^d^ gestational age (GA), ^e^ incidence rate ratio (IRR).

## 4. Future Directions

Elucidating the role of prenatal/birth characteristics and environmental exposures in RMS is essential to providing a comprehensive understanding of RMS etiology. However, compared to other pediatric cancers, these associations are highly understudied and replications of previous exposure assessments are scarce. Therefore, novel assessments are needed to understand the effects of non-genetic exposures on RMS risk. Future studies should also consider stratifying their analyses by histology and fusion status. Finally, there is a need for comparative studies that evaluate the associations of exposures and RMS risk using data from multiple countries and diverse populations. Obtaining more representative global estimates of these associations would significantly enhance the current understanding of the factors that contribute to RMS etiology.

## 5. Genetic Risk

While somatic alterations associated with RMS, such as the *PAX/FOXO1* fusion, have been well characterized, less is known about germline susceptibility to RMS. A 2015 study from our group compared 322 individuals with RMS to 322 population-based controls to evaluate whether there was an association between family history of cancer and RMS [50]. We found that individuals with a first-degree relative who had any history of cancer had an increased risk of developing ERMS (OR = 2.44, 95% CI = 1.54–3.86). Additionally, individuals with a first-degree relative that had a cancer diagnosis when they were younger than 30 years old were also at an increased risk of developing RMS (OR = 2.37, 95% CI = 1.34–4.18). These findings suggest that inherited variants in cancer predisposition genes may play a role in increasing pediatric RMS risk. To date, there have been no genome-wide association studies (GWASs) of individuals with RMS to further explore potential common genetic variations that are specific to this malignancy. 

Unlike cancers such as breast cancer or retinoblastoma, there is no Mendelian (single-gene) disorder where RMS is the primary manifestation. Thus, we review a diverse set of Mendelian disorders that have each been linked to an increased risk of RMS. Further, we discuss recently completed pediatric cancer and RMS-specific sequencing studies that have identified germline variants of interest amongst RMS patients. 

### 5.1. Autosomal Dominant Mendelian Disorders

#### 5.1.1. Li–Fraumeni Syndrome (LFS)

LFS is a cancer predisposition syndrome with an autosomal dominant inheritance pattern that is associated with deleterious germline mutations (pathogenic variants) in the *TP53* gene. These variants are reported to either inactivate the tumor suppression activity of TP53 or have a dominant negative impact [51]. Multiple different pediatric and adult-onset cancers are associated with LFS, many of which have an earlier age of onset compared to the general population.

The first report of LFS by Li and Fraumeni in 1969 included a cohort of four RMS patients [52]. Diller et al. reported three children with germline *TP53* missense mutations among 33 sporadic RMS patients [53]. Further, these three cases were all noted to be under the age of three years, indicating that germline *TP53* mutations may play a role in developing RMS earlier in life. Ognjanovic et al. also determined that children under the age of three years with a germline *TP53* mutation are at the greatest risk of developing RMS compared to other sarcomas (OR = 16.7, 95% CI = 9.4–29.7) [54]. Hettmer et al. further explored whether germline *TP53* mutations were associated with a specific RMS histological subtype [55]. They evaluated eight consecutive children with *TP53* mutations and RMS and found they all displayed the anaplastic RMS subtype. Furthermore, they evaluated seven additional patients with anaplastic RMS and unknown *TP53* status and discovered that three of these individuals harbored germline *TP53* pathogenic variants. While these findings suggest an association between anaplastic RMS and *TP53* mutations, the authors note that these conclusions should be confirmed in larger cohorts. 

#### 5.1.2. Costello Syndrome (CS)

**Figure 3:**
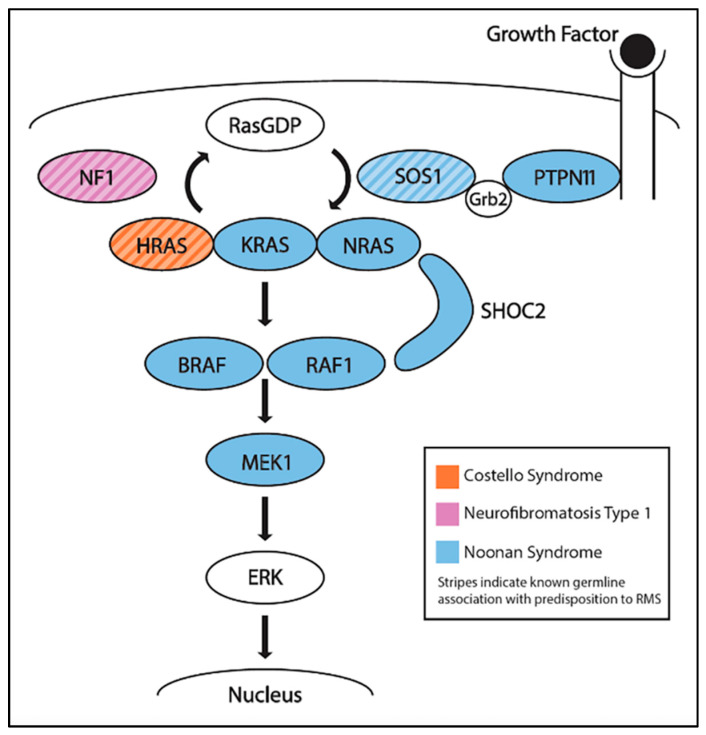
The Ras signaling pathway and proteins encoded by genes that are implicated in each of the cancer predisposition RASopathies: Costello syndrome, neurofibromatosis type I, and Noonan syndrome.

CS is a rare, developmental disorder and cancer predisposition syndrome that is caused by heterozygous activating mutations in *HRAS*. CS is one of many conditions, including Noonan syndrome and neurofibromatosis type 1, that result in the overactivation of the Ras pathway and are often referred to as RASopathies (Figure 3) [56]. Individuals with CS can have a variety of phenotypes, including significant intellectual disability, a broad neck, short stature, heart defects, and pubertal delay [57]. In a retrospective study of 29 patients with CS and a cancer diagnosis, RMS was the most commonly seen, occurring in 19 patients: 9 EMRS, 1 ARMS, 1 pleomorphic, 1 spindle-cell, 1 mixed histology, and 6 unclassified [58]. Other tumors that were detected included neuroblastoma (*n* = 5), bladder cancer (*n* = 4), and fibrosarcoma (*n* = 1). Kratz et al. later performed a retrospective analysis of cancer incidence in a larger cohort of 784 individuals with known mutations in the Ras pathway [59]. Two of twelve individuals with cancer had germline mutations in *HRAS* and developed ERMS. 

#### 5.1.3. Neurofibromatosis Type 1 (NF1)

NF1 is an autosomal dominant syndrome associated with inactivating mutations in *NF1*, a gene whose product is involved in the Ras pathway (Figure 3). NF1 includes multiple different features, including café au lait spots, benign tumors in nervous tissues (neurofibromas), optic gliomas, and learning disabilities [60]. The protein neurofibromin, encoded by *NF1*, typically negatively regulates the Ras pathway [61], thus inactivating mutations in *NF1* also in hyperactivation of the Ras pathway. Urogenital, and to a lesser extent, orbital, ERMS have been associated with NF1 [62]. Although studies have shown that the prevalence of RMS amongst NF1 patients is less than 1%, it is still greater than in individuals without NF1 [63]. In a retrospective study of 16 RMS patients with NF1, all 16 patients were identified as having ERMS, providing further evidence that patients with NF1 have a small but significant ERMS predisposition [64]. 

#### 5.1.4. Noonan Syndrome (NS)

NS is another autosomal dominant syndrome that is caused by defects in several genes in the Ras pathway, including *KRAS*, *NRAS*, *RAF1*, *BRAF*, *PTPN11*, *SOS1*, *SHOC2*, or *MEK1*, which result in Ras pathway activation (Figure 3). Individuals with NS can display developmental delay, intellectual disabilities, distinctive facial features, and congenital heart defects [65]. Despite the multiple genes that are associated with NS, the only gene with reported germline mutations in two patients with NS and ERMS is SOS1 [66,67]. In a study of 45 individuals with NS and a history of cancer, the top three malignancies were ALL (*n* = 8), neuroblastoma (*n* = 8), and RMS (*n* = 6, 1 botryoid and 5 ERMS) [58]. 

#### 5.1.5. DICER1 Tumor Syndrome

DICER1 tumor syndrome, also known as pleuropulmonary blastoma familial tumor susceptibility syndrome, is an autosomal dominant disorder resulting from germline inactivating mutations of *DICER1*. DICER1 is a miRNA processing enzyme, and as such, broadly influences gene expression [68]. Individuals with mutations in *DICER1* are at an increased risk of developing RMS and many other cancer types, including pleuropulmonary blastoma (the highest cancer risk), cystic nephroma, thyroid cancer, and ovarian Sertoli–Leydig cell tumors [69]. RMS tumors that are associated with *DICER1* mutation often arise in the cervix [70]. In these individuals, the somatic variant (second hit) in the tumor is frequently a missense variant in the RNAse III domain [71]. Due to the association of RMS with germline loss-of-function *DICER1* mutations, Doros et al. investigated the prevalence of somatic *DICER1* mutations in a cohort of 52 sporadic, unselected ERMS patients that did not have DICER1 tumor syndrome [72]. They found that two of the 52 sporadic cases displayed *DICER1* mutations in the tumors, one of which was a missense mutation that was predicted to interrupt a splice site and the other was an in-frame deletion of a glutamic acid residue near the RNAse III domains. There was no functional assessment of these mutations, but both were predicted to negatively impact DICER1 function. These findings strengthened the putative association between DICER1 syndrome and increased RMS risk. Stewart et al. surveyed three cohorts of individuals with germline mutations in *DICER1* to determine the incidence of neoplasms [73]. Their study revealed six RMS patients among the 148 probands, and RMS was the fourth most commonly detected neoplasm amongst the *DICER1* mutation carriers, behind pleuropulmonary blastoma (*n* = 47), Sertoli–Leydig cell tumor (*n* = 24), and thyroid cancer (*n* = 10). The mean age of diagnosis of the six RMS patients was 10 years old.

#### 5.1.6. Rubinstein–Taybi Syndrome (RTS)

RTS is an autosomal dominant disorder that is caused by inactivating mutations, primarily in *CREBBP*, and to a lesser degree, *EP300*. Individuals with RTS have multiple congenital anomalies, including broad thumbs and dysmorphic facial features [65]. An early case report from 1981 described a 4-year-old nasopharyngeal RMS patient with RTS [74]. In a more recent study of more than 700 patients with RTS, two further individuals were identified that had nasopharyngeal RMS [75]. 

#### 5.1.7. Retinoblastoma (RB)

Hereditary RB is an autosomal dominant syndrome that is caused by inactivating mutations in *RB1*. Approximately 90% of individuals with a germline mutation in *RB1* will develop retinoblastoma [76]. After the treatment and eradication of the initial retinoblastoma, these individuals can also develop a number of secondary cancers later in life. RMS, especially ERMS, is occasionally detected as one such secondary neoplasm in individuals with *RB1* mutations that have completed retinoblastoma treatment [77]. Temming et al. analyzed 648 retinoblastoma patients from 1940–2008 to determine whether their treatment course (radiotherapy or chemotherapy) influenced the incidence of secondary cancers [78]. They found that soft-tissue sarcomas (including RMS) were the most commonly seen secondary cancers seen in inherited retinoblastoma (RB1) survivors, and that radiotherapy significantly increased the risk of secondary cancer in retinoblastoma patients (HR = 2.97, 95% CI = 1.64–5.36). Kleinermann et al. evaluated 963 survivors (one-year post-diagnosis) of hereditary retinoblastoma to calculate the risk of developing a second soft-tissue sarcoma amongst these individuals [79]. They reported a total of 69 sarcoma diagnoses amongst 68 patients, 8 of which were RMS. They calculated that retinoblastoma survivors were at a significantly increased risk of developing RMS (SIR = 279, 95% CI = 120–551). An example of an RMS transition after radiotherapy treatment was described by Cebulla et al. in a patient who presented with retinoblastoma, received radiotherapy, and subsequently developed RMS in the field of radiation within 2 years of treatment [80].

### 5.2. Autosomal Recessive Mendelian Disorders

#### 5.2.1. Constitutional Mismatch Repair Deficiency (CMMRD)

CMMRD is an autosomal recessive disorder that is caused by biallelic mutations in one of the DNA mismatch repair genes: *MLH1*, *MSH2*, *MSH6*, and *PMS2*. Individuals with CMMRD may display café au lait spots, similar to individuals with NF1. They also have a high risk of developing a wide variety of malignancies in childhood [81]. In 2015, Lavoine et al. performed a retrospective study of 31 CMMRD patients with a history of cancer [82]. Although CNS tumors, leukemias, and gastrointestinal tumors predominated, three individuals were diagnosed with sarcomas (one dermatofibrosarcoma and two osteosarcoma). In another study, two patients from consanguineous families with CMMRD developed RMS [83]. Overall, it has been reported that 10–15% of CMMRD patients present “non-canonical” tumors, including soft-tissue sarcomas, such as RMS [81]. 

#### 5.2.2. Fanconi Anemia (FA)

FA is an autosomal recessive disorder that is characterized by biallelic mutations in the *FANC* family of genes. Individuals with FA have a variety of hematological problems, including frequent development of acute myelogenous leukemia [84]. A retrospective study of 10 pediatric patients with FA and a history of solid tumors identified two patients with RMS, one of which had ERMS [85]. Human xenograft assays have shown that in fusion-positive ARMS, inhibition of the *FANCD2* gene may represent a potentially viable treatment avenue [86].

#### 5.2.3. Mosaic Variegated Aneuploidy Syndrome (MVA)

MVA is a rare autosomal recessive disorder that is characterized by consistent gain or loss of chromosomes (often monosomy or trisomy) in cells throughout a variety of tissues. Those affected with MVA typically display growth deficiencies, microcephaly, and an increased risk of developing pediatric cancer [87]. Hanks et al. surveyed five families with a history of MVA, two of which had instances of RMS: a 7-year-old with ERMS of the soft palate and a 5-month-old with ERMS of the vagina. Both of these patients had biallelic deleterious or loss-of-function mutations in the *BUB1B* gene, the first of several genes associated with MVA [88]. BUB1B protein plays a critical role in the mitotic spindle checkpoint.

### 5.3. Epigenetic Mechanisms

#### Beckwith–Wiedemann Syndrome (BWS)

BWS is caused by a complex mechanism of epigenetic silencing of regions of chromosome 11. Individuals with BWS display overgrowth phenotypes and have an increased risk of developing multiple cancers, including Wilms tumor, hepatoblastoma, neuroblastoma, and RMS [89]. Smith et al. reported three patients with BWS and RMS, all of whom had fusion-negative ARMS [90]. They note these findings were a departure from earlier case reports [91,92,93], which suggested that RMS in BWS may only be associated with ERMS. More recently, Mussa et al. performed a pooled analysis of 1370 BWS patients to determine cancer incidence and whether certain BWS epigenetic changes had different cancer phenotypes [94]. While Wilms tumor (*n* = 48) and hepatoblastoma (*n* = 23) were the two most common cancers detected, they also described seven patients with RMS amongst the pooled cohorts, six of whom shared the same epigenetic change, a loss of methylation of *ICR2*. They documented that patients with BWS have an increased risk of RMS compared to the general population. A follow-up pooled analysis from Maas et al. added a further 229 BWS patients and detected an additional RMS case [95].

## 6. Large-scale Pediatric Cancer Germline Genetics Studies

Recent studies of larger cohorts of unselected pediatric cancer patients have both identified germline findings relevant to RMS predisposition, as well as somatic mutations that are associated with the previously discussed cancer predisposition syndromes.

Our group completed the Baylor Advancing Sequencing in Childhood Cancer Care (BASIC3) study, which performed paired clinical germline (normal) and somatic (tumor) whole-exome sequencing (WES) on newly diagnosed pediatric cancer patients with solid tumors [96]. Of the first 150 patients, there were 15 patients diagnosed with RMS. There was one germline finding, namely, a patient with a *TP53* germline mutation. Three further patients had somatic mutations in genes related to cancer predisposition syndromes, namely, *DICER1*, *HRAS* (Costello), and *PTPN11* (Noonan).

In a study from St. Jude Research Hospital, Zhang et al. reviewed whole-genome and whole-exome sequencing on 1120 pediatric cancer patients to determine the prevalence of germline mutations in known cancer predisposition genes [97]. Among the 43 RMS patients assessed, they reported germline mutations in three individuals: two in *TP53* (Li–Fraumeni Syndrome) and one in *BRCA2. BRCA2* belongs to the Fanconi anemia family of genes and is well-known for its association with adult-onset cancer, including breast and ovarian cancer. Although this was the first report of heterozygous *BRCA2* mutations and RMS, more recent studies (see below) have strengthened the evidence of this relationship.

In the Peds-MiOncoSeq effort at the University of Michigan, Mody et al. performed germline and tumor WES and transcriptomic analysis on 102 children and young adults with relapsed or rare cancers in an effort to identify mutations in these individuals that may be clinically actionable [98]. They identified two RMS patients with germline findings: one with a frameshift *FANCD2* mutation (FA) and another with a stopgain *TP53* (LFS) mutation.

### Large-Scale RMS-Specific Studies

Although RMS is associated with a variety of cancer predisposition syndromes (as described above), it is not the primary tumor type observed in any disorder. RMS-specific sequencing studies can address this gap in knowledge and identify whether there are genes of interest that are specific to RMS. Two such RMS-specific cohort sequencing studies have recently explored both the frequency and identity of germline mutations in cancer predisposition genes in RMS patients. Our group completed the Genetics of Embryonal and Alveolar Rhabdomyosarcoma Study (GEARS), which performed germline WES analysis of 615 RMS patients from the Children’s Oncology Group in order to determine the prevalence of germline mutations in 70 known cancer predisposition genes [99]. We found that 45 of the 615 patients (7.3%) possessed cancer predisposition germline mutations. ERMS was more common than ARMS in these individuals, and all of this group had fusion-negative RMS. Those with a germline mutation were more likely to be younger at diagnosis. The most common germline findings in CPGs that are known to be associated with RMS were in *TP53* (LFS; *n* = 11), *NF1* (*n* = 9), and *HRAS* (CS; *n* = 5). There were 12 patients with germline mutations in cancer predisposition genes that are not currently associated with RMS, including two genes with heterozygous mutations in more than one patient: *BRCA2* (*n* = 6) and *SDHA* (*n* = 2). The enrichment of germline variants in *BRCA2* among RMS patients compared to a control population was significant (OR = 3.55, 95% CI = 1.43–9.40). There were no patients with biallelic mutations consistent with a recessive syndrome diagnosis.

A second study from Kim et al. used whole-genome sequencing (WGS) to determine the rate and identity of germline mutations in a cohort of 273 intermediate-risk RMS patients from the COG [100]. They also performed WES on a secondary cohort, unselected for risk, that comprised 121 RMS patients from the COG, the Cooperative Human Tissue Network, the Children’s Hospital at Westmead, and NCI for further comparison. Among both cohorts, there were individuals with mutations in RMS-associated cancer predisposition genes, including *TP53*, *DICER1*, *NF1*, *MSH2*, and *MSH6*. This study reported one individual with a heterozygous germline mutation in *BRCA2* and another with biallelic germline mutations in *BRCA2* consistent with Fanconi anemia D2. In this study, germline mutations were also detected in genes that are rarely reported with RMS, including single individuals with an *ERCC2*, *CBL*, *SMARCA4*, *RET*, and *RH* mutation. Whether these five newly reported genes play a role in RMS is unknown and replication studies are needed.

## 7. Future Directions

RMS-specific studies are needed to address the lack of knowledge of RMS susceptibility genes and pathogenic variants. A GWAS of RMS may further identify common variants and novel genes that are associated with an increased risk of developing this tumor. Additionally, exome and/or genome sequencing is emerging as a clinically relevant tool for identifying novel genes in pediatric cancer. To build upon the recent RMS sequencing studies, analysis of larger sets of sarcoma-related genes and the integration of techniques to increase the power of detection (e.g., burden testing) is needed. As more germline susceptibility variants are discovered using these methods, the identification of regulatory variants that cooperate with somatic driver mutations would greatly enhance the approach to multidisciplinary, integrated studies of RMS. This has been a notable area of study in Ewing sarcoma [101,102].

Furthermore, an analysis of the different subtypes of RMS may identify whether certain predisposition syndromes are specifically associated with one specific, or a few, RMS subtypes. For example, in medulloblastoma (MB), children with certain inherited cancer syndromes will present with specific MB subtypes: individuals with Gorlin syndrome have an increased risk to develop Sonic Hedgehog (SHH)-MB; conversely, individuals with familial adenomatous polyposis syndrome are at risk of developing the WNT-MB subtype [103]. Discovering new associations and further studying purported associations will further our understanding of subtype-specific RMS predisposition including lesser-known subtypes, such as pleomorphic and spindle-cell/sclerosing RMS.

To date, RMS has been modeled in human cell lines, mice, and zebrafish. Fusion-positive RMS has been modeled in mouse mesenchymal stem cells [104], as well as human fetal skeletal muscle cells [105]. Fusion-negative RMS has been modeled in zebrafish through the over-activation of the Ras pathway [106]. These cellular and non-human models can similarly be used in the future to further explore both known CPGs for disease mechanisms, as well as novel CPGs derived from GWASs or sequencing studies.

Additionally, studies of the role of genetic variants in cancer outcomes could generate important clinical knowledge. Although the identification of pathogenic germline variants in rare diseases, such as RMS, is challenging, studies of other childhood cancers have shown that pathogenic germline variants may contribute to adverse outcomes. Studies have shown that germline variants in *GATA3* and *TP53* may predispose children with ALL to outcomes such as secondary malignancies and relapse [107,108,109]. Further, a pan-cancer analysis of childhood cancer revealed that of the 6% of patients who harbored a pathogenic germline variant in a CPG, 3% also carried germline alterations that were considered potentially druggable events (defined as variants in genes with direct or indirect targeted therapy that is currently available or in development). As awareness of the importance of germline genetics for the therapeutic development of cancer increases [110], there are opportunities to integrate the knowledge of germline variants with known somatic targets for RMS patients with particularly poor or severe outcomes.

## 8. Conclusions

As the breadth of knowledge of the individual effects of environmental exposures and genetic variants on RMS predisposition increases, a significant step for understanding the disease etiology would be to examine the gene–environment interactions that may also contribute to this complex disease. To our knowledge, there have been no published studies of gene–environment interactions in RMS, though there have been some reports in other pediatric cancers [111]. Ultimately, there is a need for increased efforts for both environmental and genetic studies in order to translate findings into clinical practice. Confirming the environmental exposures that play a role in RMS could inform preventative strategies, primarily regarding maternal health and in utero exposures. Additionally, identifying novel RMS-associated genes and germline variants will aid in enhancing risk stratification, developing therapeutic targets, and addressing poor outcomes.
